# New 1*H*-Benzo[*f*]indazole-4,9-diones Conjugated with C-Protected Amino Acids and Other Derivatives: Synthesis and *in Vitro* Antiproliferative Evaluation

**DOI:** 10.3390/molecules201219809

**Published:** 2015-12-08

**Authors:** Aurora Molinari, Alfonso Oliva, Marlene Arismendi-Macuer, Leda Guzmán, Mauricio Fuentealba, Marcela Knox, Raúl Vinet, Arturo San Feliciano

**Affiliations:** 1 Instituto de Química, Facultad de Ciencias, Pontificia Universidad Católica de Valparaíso, Valparaíso 2373223, Chile; m.arismendi.m@gmail.com (M.A.-M.); leda.guzman@ucv.cl (L.G.); mauricio.fuentealba@ucv.cl (M.F.); marcela.knox@gmail.com (M.K.); 2 Facultad de Farmacia, Universidad de Valparaíso, Valparaíso 2360102, Chile; raul.vinet@uv.cl; 3 Centro Regional de Estudios en Alimentos y Salud (CREAS), Valparaíso 2362696, Chile; 4 Facultad de Farmacia, Departamento de Química Farmacéutica, CIETUS, IBSAL, Universidad de Salamanca, Salamanca 37007, Spain; asf@usal.es

**Keywords:** 1,4-naphthoquinone, 1*H-*benzoindazole, pyrazole, amino acid

## Abstract

1*H*-Benzo[*f*]indazole-4,9-dione derivatives conjugated with C-protected amino acids (glycine, l-alanine, l-phenylalanine and l-glutamic acid) **6a**–**l** were prepared by chemically modifying the prenyl substituent of 3-methyl-7-(4-methylpent-3-enyl)-1*H*-benzo[*f*]indazole-4,9-dione **2** through epoxidation, degradative oxidation, oxidation and N-acyl condensation reactions. The chemical structures of the synthesized compounds were elucidated by analyzing their IR, ^1^H-NMR and ^13^C-NMR spectral data together with elemental analysis for carbon, hydrogen and nitrogen. The preliminary *in vitro* antiproliferative activity of the synthesized derivatives was evaluated on KATO-III and MCF-7 cell lines using a cell proliferation assay. The majority of the derivatives exhibited significant antiproliferative activity with IC_50_ values ranging from 25.5 to 432.5 μM. These results suggest that 1*H*-benzo[*f*]indazole-4,9-dione derivatives are promising molecules to be researched for developing new anticancer agents.

## 1. Introduction

A considerable number of naturally occurring and synthetic compounds that contain a 1,4-quinone moiety have been investigated for antitumor activity [1,2,3,4,5]. These compounds generate a quinone/hydroquinone one-electron redox process that inhibits mitochondrial electron transport and decouples oxidative phosphorylation [5]. Additionally, they act as topoisomerase inhibitors via DNA intercalation or as alkylating agents that add across both strands of the double helix, thereby leading to cancer cell death. Furthermore, it has been suggested that the quinone-induced inhibition of cancer cell growth can be attributed to the generation of reactive oxygen species (ROS) after redox cycling [1,2,3,4,5,6,7]. 1*H*-Indazolediones are nitrogen-containing heterocyclic 1,4-quinones that possess interesting chemical and biological properties, including antitumor activities against Ehrlich ascites carcinoma growth in male CF_1_ mice, P-388 lymphocytic leukemia in male BDF_1_ mice and L1210 murine leukemia cells [8,9,10,11,12,13,14,15,16,17,18]. In the literature, there are various synthetic methods for preparing 1*H*-indazoles [8], whereas 1*H*-indazole-4,7 and 4,9-diones are synthesized via the 1,3-dipolar cycloaddition of diazomethanes to 1,4-quinones [10,19,20,21,22]. We have recently reported the synthesis of unsubstituted and *N*-substituted 3-methyl-7-(4-methylpent-3-enyl)-1*H*-benzo[*f*]indazole-4,9-diones **2** from the reaction of 2-acetyl-6-(4-methylpent-3-enyl)-1,4-naphthoquinone **1** with hydrazine or substituted hydrazines [23]. The objective of this work was to continue previous research on the design and synthesis of new potentially cytotoxic 1,4-naphthoquinone compounds [24,25] while taking into account the enhanced cytotoxic effect that has been observed in drugs or compounds conjugated with amino acids [26,27]. Therefore, we prepared twenty one new 1*H*-benzo[*f*]indazole-4,9-dione compounds conjugated with glycine and the l-type amino acids alanine, phenylalanine, and glutamic acid **6a**–**l**, as well as the epoxides **3a**–**c**, aldehydes **4a**–**c** and carboxylic acids **5a–c**, by chemically modifying the prenyl 7-(4-methylpent-3-enyl) substituent of **2**. Additionally, we evaluated the antiproliferative activity of these new compounds on KATO-III and MCF-7 cell lines of human gastric cancer and human breast cancer, respectively.

## 2. Results and Discussion

### 2.1. Chemistry

The new derivatives were prepared using 1*H*-benzo[*f*]indazole-4,9-diones **2** (R = -H; -CH_2_CH_2_OH; -CH_2_CH_2_OAc) as starting substrates, which were conveniently obtained through a direct cyclization reaction of 2-acetyl-6-(4-methylpent-3-enyl)-1,4-naphthoquinone **1** with hydrazines, triethylamine and catalytic glacial acetic acid [23]. The first step of this reaction could be (i) the conjugate addition of the hydrazine to the 1,4-naphthoquinone unit to afford the 3-substituted 1,4-hydroquinone compound **1’** or (ii) the condensation reaction between the acetyl group and hydrazine to form the corresponding hydrazone **1T’**.

**Scheme 1:**
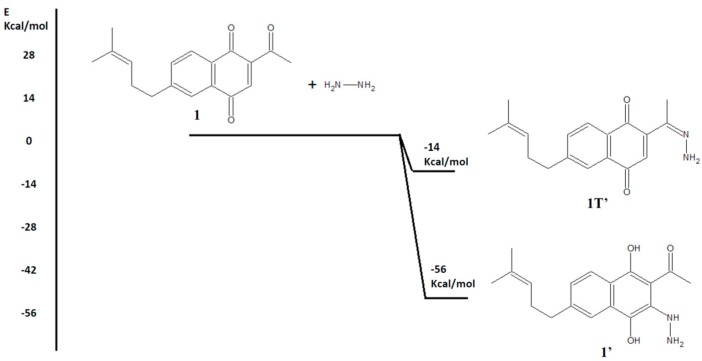
Comparison between reaction energies of **1** to **1’** and **1T’**.

**Figure 1:**
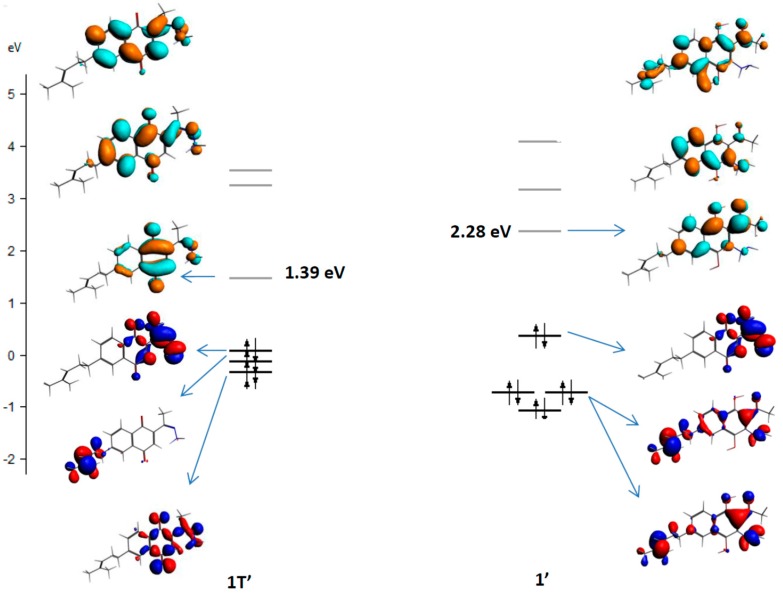
Molecular orbital diagrams of compounds **1T’** and **1’**. The HOMO energies have been arbitrarily set to zero for clarity.

To rationalize the possible products, we performed full geometry optimizations of the structures using preliminary density functional theory (DFT) calculations (see the Experimental Section). With the aim of exploring the reactants and possible products of the first step of this reaction from a thermodynamics perspective, we subtracted the total bonding energies of the products from the total bonding energies of the reactants. The comparison between these energy changes indicated that the formation of **1’** is 4-fold more favorable than the formation of **1T’** (Scheme 1). Moreover, the HOMO-LUMO gaps of compounds **1’** and **1T’** were 2.28 eV and 1.39 eV, respectively, which indicates that the most stable derivative is compound **1’** (Figure 1). These results suggest that cyclization must initially occur by the conjugate addition to afford the 3-substituted 1,4-hydroquinone compound **1’**, but a complete transition state (TS) search and intrinsic reaction coordinate (IRC) study are needed to confirm this hypothesis. For this reason, our research group is currently working to obtain a full reaction pathway for all reaction steps.

**Scheme 2:**
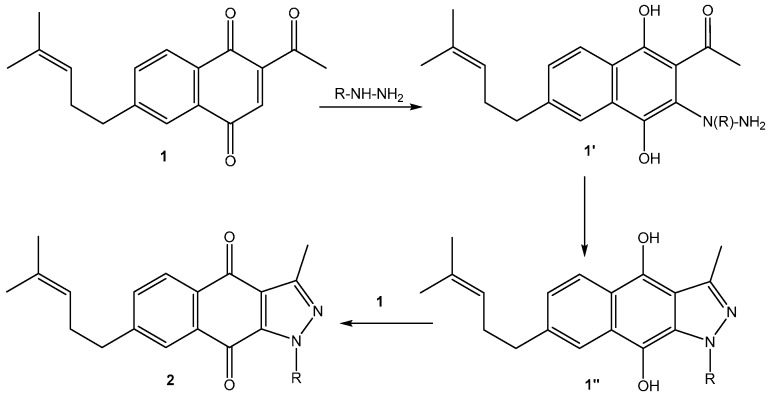
Cyclization pathway for the *N*-substituted 1*H*-benzo[*f*]indazole-4,9-diones **2**.

The second step of the reaction is the cyclization of the 3-substituted 1,4-hydroquinone compound **1’** by the nucleophilic addition/elimination reaction between the amino and carbonyl groups of **1’** to afford the fused pyrazolo-1,4-naphthohydroquinone compound **1’’**. Finally, this compound is oxidized to the 1,4-naphthoquinone **2** by the initial 2-acetyl-1,4-naphthoquinone **1** (Scheme 2), a step that is supported by the isolation of 2-acetyl-6-(4-methylpent-3-enyl)-1,4-naphthohydroquinone as a by-product [10,28].

**Scheme 3:**
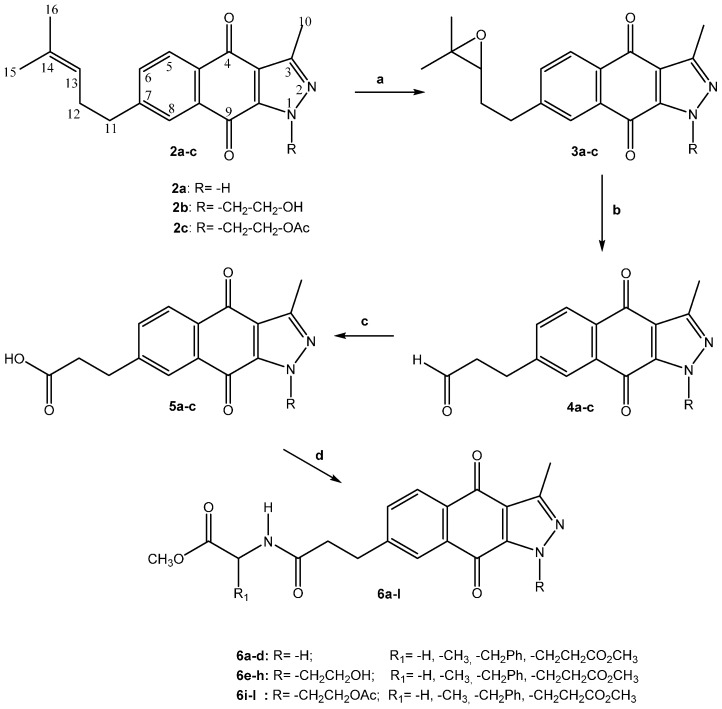
Synthetic pathway for the new conjugated derivatives **6a**–**l**. **(a)** mCPBA, CH_2_Cl_2_, NaHCO_3_, rt, 4 h; **(b)** H_5_IO_6_, THF, H_2_O, rt, 2 h; **(c)** NaClO_2_, NaH_2_PO_4_, H_2_O, *t*-BuOH, 2-methyl-2-butene, rt, 27 h; **(d)** EtOCOCl, Et_3_N, THF, 20 min, 0 °C, R_1_CH(NH_2_)CO_2_Me, rt, 16 h.

To synthesize the new 1*H*-benzo[*f*]indazole-4,9-diones conjugated with C-protected amino acids **6a–l**, we followed the synthetic pathway shown in Scheme 3. The epoxidation of the double bond in the 7-(4-methylpent-3-enyl) group of **2a**–**c** to afford oxiranyl compounds **3a**–**c** was accomplished with *m*-chloroperoxybenzoic acid (mCPBA), and treatment of these compounds with periodic acid afforded the aldehydes **4a**–**c** [29]. Oxidation of these aldehydes to the carboxylic acids **5a**–**c** was performed with sodium chlorite in the presence of a catalytic amount of 2-methyl-2-butene. The reactivity of the carboxylic group was then enhanced through the *in situ* formation of the mixed anhydride with ethyl chloroformate followed by the addition of the corresponding methyl ester of glycine, l-alanine, l-phenylalanine and l-glutamic acid [25]. In all of these synthesized compounds, the l-configuration must be retained in the amino acid unit. The physical and analytical data of the compounds are presented in the experimental section along with the IR, ^1^H and ^13^C spectroscopic data; chemical shifts are reported according to the carbon numbering of compounds **2** in Scheme 3.

The common features from the spectral data of compounds **6a**–**l** are closely related to those previously reported for the starting compounds **2a**–**c** [23], and they are as follows:

- In some cases, their IR spectra show two carbonyl-quinone absorptions at approximately 1680 and 1670 cm^−1^, but the latter absorption is primarily observed.
- In the ^1^H spectra, the singlet of the C-10 methyl group appears at approximately 2.60 to 2.80 ppm, the coupled methylene groups of C-11 and C-12 carbons show triplets or multiplets between 2.50 and 3.00 ppm (*J* = 7.3–8.0 Hz), and the coupled aromatic hydrogen of carbon C-5, C-6 and C-8 are observed as doublets of doublets and two doublets at 7.70 to 8.10 ppm (*J* = 7.6 and 1.6 Hz).
- The ^13^C-NMR spectra contain signals for carbonyl-quinone C-4 and C-9 carbon atoms at 170 to 180 ppm.

### 2.2. Biological Assay

The antiproliferative activity of the synthesized compounds was assessed on KATO-III and MCF-7 cell lines using a CellTiter 96^®^ AQueous One Solution Proliferation Assay (MTS) from Promega (Madison, WI, USA) with doxorubicin as a control. The results were expressed as the concentration determining 50% inhibition of cell proliferation (IC_50_).

Table 1 and Table 2 show the IC_50_ values for the antiproliferative activity obtained for each derivative tested in KATO-III and MCF-7 cell lines, respectively. Each column in Table 1 and Table 2 contains the IC_50_ values for derivatives belonging to Series-I, -II and -III, respectively.

The antiproliferative activity determined in KATO-III cell lines for Series-I, -II and -III of 1*H*-benzo[*f*]indazole-4,9-dione-based derivatives ranged from 60.3 (**2a**) to 326.6 μM (**5a**), 25.5 (**2b**) to 401.8 μM (**3b**), and 33.0 (**2c**) to 324.3 (**3c**), respectively (Table 1). Similarly, the antiproliferative activity assayed in MCF-7 cell lines for Series-I, -II and -III 1*H*-benzo[*f*]indazole-4,9-dione-based derivatives ranged from 63.2 (**2a**) to 432.5 μM (**3a**), 27.5 (**2b**) to 415.9 μM (**3b**), and 29.4 (**2c**) to 389.9 μM (**3c**), respectively (Table 2).

By comparing Table 1 and Table 2 is possible to observe the similarity between the patterns generated from the IC_50_ values obtained from Series-I, -II and -III derivatives. Moreover, these patterns were very similar in both cell models.

**Table 1:** *In vitro* antiproliferative activities of 1*H*-benzo[*f*]indazole-4,9-dione-based derivatives expressed as IC_50_ values obtained in KATO-III cell line.

Series-I	Series-II	Series-III
IC_50_ (CI 95%) μM	IC_50_ (CI 95%) μM	IC_50_ (CI 95%) μM
*p*	*p*	*p*
**2a**	**2b**	**2a**
60.3 (18.9–192.4)	25.5 (9.4–69.1)	33.0 (8.0–136.5)
C: NA, R: NA	C: NA, R: NS	C: NA, R: NS
**3a**	**3b**	**3c**
313.3 (110.4–889.3)	401.8 (166.8–967.8)	324.3 (132.9–791.3)
C: ***, R: NA	C: ****, R: NS	C: ****, R: NS
**4a**	**4b**	**4c**
99.5 (50.1–197.7)	63.0 (22.4–176.9)	60.5 (29.8–123.1)
C: ***, R: NA	C: NS, R: NS	C: NS, R: NS
**5a**	**5b**	**5c**
326.6 (167.9–635.4)	337.3 (192.3–591.5)	162.6 (70.6–374.2)
C: ***, R: NA	C: ****, R: NS	C **, R: NS
**6a**	**6e**	**6i**
230.7 (82.9–642.2)	310.5 (132.5–727.4)	208.0 (92.5–467.6)
C: **, R: NA	C: ****, R: NS	C: ***, R: NS
**6b**	**6f**	**6j**
114.8 (57.7–228.6)	43.5 (15.9–118.4)	54.1 (13.6–215.1)
C: NS, R: NA	C: NS, R: *	C: NS, R: NS
**6c**	**6g**	**6k**
126.8 (41.3–389.4)	37.6 (11.5–123.4)	34.9 (16.7–72.9)
C: NS, R: NA	C: NS, R: *	C: NS, R: **
**6d**	**6h**	**6l**
111.7 (35.3–353.1)	52.8 (16.8–166.7)	109.6 (32.5–370.4)
C: NS, R: NA	C: NS, R: NS	C: NS, R: NS

The results are presented as means and 95% confidence intervals (CI 95%) for three independent experiments. C and R indicate column and row, respectively. *, **, *** and **** indicate significant differences at *p* < 0.05, 0.01, 0.001 and 0.0001, respectively. NA and NS indicate not available and not significant, respectively. Doxorubicin exhibited an IC_50_ of 4.0 μM (0.9–17.4) μM in KATO-III cell line.

**Table 2:** *In vitro* antiproliferative activities of 1*H*-benzo[*f*]indazole-4,9-dione-based derivatives expressed as IC_50_ values obtained in MCF-7 cell line.

Series-I	Series-II	Series-III
IC_50_ (CI 95%) μM	IC_50_ (CI 95%) μM	IC_50_ (CI 95%) μM
*p*	*p*	*p*
**2a**	**2b**	**2c**
63.2 (24.8–161.5)	27.5 (71.1–106.8)	29.4 (14.1–61.3)
C: NA, R: NA	C: NA, R: NS	C: NA, R: NS
**3a**	**3b**	**3c**
432.5 (167.8–1115.1)	415.9 (202.0–856.2)	389.9 (222.2–684.4)
C: ****, R: NA	C: ****, R: NS	C: ****, R: NS
**4a**	**4b**	**4c**
123.6 (39.6–385.7)	43.4 (18.6–101.1)	33.0 (10.8–100.7)
C: NS, R: NA	C: NS, R: *	C: NS, R: **
**5a**	**5b**	**5c**
372.4 (185.1–749.2)	335.0 (186.7–601.1)	244.9 (95.0–631.0)
C: ***, R: NA	C: ****, R: NS	C: ****, R: NS
**6a**	**6e**	**6i**
413.0 (138.2–1234.7)	291.1 (155.1–546.2)	255.3 (115.8–562.8)
C: ****, R: NA	C: ****, R: NS	C: ****, R: NS
**6b**	**6f**	**6j**
94.2 (44.8–197.9)	62.7 (17.0–231.2)	52.6 (24.2–114.2)
C: NS, R: NA	C: NS, R: NS	C: NS, R: NS
**6c**	**6g**	**6k**
154.9 (59.2–405.2)	39.0 (11.6–131.1)	35.4 (8.4–149.5)
C: NS, R: NA	C: NS, R: **	C: NS, R: ***
**6d**	**6h**	**6l**
143.9 (60.3–343.5)	87.9 (45.0–171.7)	99.8 (52.2–190.6)
C: NS, R: NA	C: NS, R: NS	C: NS, R: NS

The results are presented as means and 95% confidence intervals (CI 95%) for three independent experiments. C and R indicate column and row, respectively. *, **, *** and **** indicate significant differences at *p* < 0.05, 0.01, 0.001 and 0.0001, respectively. NA and NS indicate not available and not significant, respectively. Doxorubicin exhibited an IC_50_ of 0.3 μM (0.3–1.3) μM in MCF-7 cell line.

A closer analysis using a two-way ANOVA test followed by a Dunnett’s multiple comparison post-test showed that the most promising derivatives were compounds **2** (*i.e*., **2a**, **2b** and **2c**), compounds **4** (*i.e*., **4a**, **4b** and **4c**) and derivatives conjugated with l-alanine (*i.e*., **6b**, **6f** and **6j**), l-phenylalanine (*i.e.*, **6c**, **6g** and **6k**) and l-glutamic acid (*i.e*., **6d**, **6h** and **6l**). Additionally, the statistical analysis shows that the compounds of Series-II and -III have better IC_50_ values compared to compounds of Series-I.

## 3. Experimental Section

### 3.1. Chemistry

#### 3.1.1. General

All reactions were performed using reagents and solvents purchased from commercial sources and purified by standard procedures as necessary. Starting *N*-substituted 1*H*-benzo[*f*]indazole-4,9-diones **2a**–**c** were synthesized according to a previously described procedure [23]. IR spectra were recorded on a Perkin Elmer FT IR 1600 spectrophotometer (Norwalk, CN, USA) as a film over NaCl discs. NMR spectra were recorded on a Bruker Avance 400 Digital NMR spectrometer (Bruker/Analytic, Karlsruhe, Germany) operating at 400.13 MHz for ^1^H and 100.62 MHz for ^13^C in CDCl_3_, acetone-d_6_ or DMSO-*d*_6_ with internal TMS as a reference. Chemical shifts (δ) were expressed in ppm, followed by multiplicity and coupling constant (*J*) in Hz. Elemental analyses of C, H and N were performed using a Perkin Elmer 2400 Series II CHN Elemental Analyzer (Perkin Elmer Inc., Waltham, MA 02451, USA). The reaction progress was monitored by thin layer chromatography with Silica gel 60 F_254_ (0.25 mm thick, Merck, Darmstadt, Germany) aluminum sheets, whereas column chromatographies were performed on Silica gel 60 (230–400 mesh, Merck) using solvent mixtures with variable proportions as eluents. Melting points were determined on a Stuart SMP 10 apparatus (Stone, Staffordshire, UK), and they were not corrected.

#### 3.1.2. General Procedure for the Preparation of 7-[2-(3,3-Dimethyloxiranyl)-ethyl]-3-methyl-1*H*-benzo[*f*]indazole-4,9-diones **3a**–**c**

The compounds were synthesized by mCPBA (9.5 mmol) epoxidation of the 1*H*-benzo[*f*]indazole-4,9-dione **2a**–**c** (9.5 mmol) and 1.34 g of NaHCO_3_ in CH_2_Cl_2_ (250 mL) at rt for 2 h under agitation. The crude epoxide was purified by column chromatography with *n*-hexane/ethyl acetate as the eluent.

*7-[2-(3,3-Dimethyloxiranyl)-ethyl]-3-methyl-1H-benzo[f]indazole-4,9-dione* (**3a**): This compound was prepared following the general procedure from 3-methyl-7-(4-methylpent-3-enyl)-1*H-*benzo[*f*]indazole-4,9-dione **2a**. Light orange solid purified with 1:1 hexane/ethyl acetate, 84% yield, m.p. 134–136 °C; IR (NaCl, ν/cm^−1^) 3140 (NH), 1668 (C=O). ^1^H-NMR (CDCl_3_) δ 1.16 (s, 3H, CH_3_, H16), 1.28 (s, 3H, CH_3_, H15), 1.83–1.90 (m, 2H, CH_2_, H12), 2.75 (s, 3H, CH_3_, H10), 2.80 (t, *J* = 7.4 Hz, 1H, CH, H13), 2.87 (t, *J* = 7.4 Hz, 2H, CH_2_, H11), 7.97 (dd, *J*_1_ = 7.9 Hz, *J*_2_ = 1.6 Hz, 1H, CH, H6), 8.06 (d, *J* = 1.6 Hz, 1H, CH, H8), 8.15 (d, *J* = 7.9 Hz, 1H, CH, H5), 13.9 (s, 1H, NH, R). ^13^C-NMR (CDCl_3_) δ 11.7, 18.6, 24.7, 30.1, 32.8, 58.7, 63.5, 118.2, 127.0, 127.5, 128.2, 129.7, 130.2, 133.5, 134.0, 147.8, 178.3, 180.1. Elemental analysis calcd for C_18_H_18_N_2_O_3_: C 69;77; H 5.84; N 9.03; found: C 67.99; H 5.88; N 8.94.

*7-[2-(3,3-Dimethyloxiranyl)-ethyl]-1-(2-hydroxyethyl)3-3-methyl-1H-benzo[f]indazole-4,9-dione* (**3b**): This compound was prepared following the general procedure from 1-(2-hydroxy-ethyl)-3-methyl-7-(4-methylpent-3-enyl)-1*H-*benzo[*f*]indazole-4,9-dione **2b**. Brown solid purified with 1:1 hexane/ethyl acetate, 74% yield, m.p. 62–64 °C; IR (NaCl, ν/cm^−1^) 3330 (OH), 1670, 1658 (C=O). ^1^H-NMR (CDCl_3_) δ 1.05 (s, 3H, CH_3_, H16), 1.26 (s, 3H, CH_3_, H15), 1.72–1.84 (m, 2H, CH_2_, H12), 2.44 (s, 3H, CH_3_, H10), 2.73 (t, *J* = 6.4 Hz, 1H, CH, H13), 2.84 (t, *J* = 6.4 Hz, 2H, CH_2_, H11), 3.77 (t, *J* = 5.8 Hz, 2 H, CH_2_N, R), 4.55 (t, *J* = 5.8 Hz, 2 H, CH_2_O, R), 4.90 (s, 1H, OH, R), 7.67 (dd, *J*_1_ = 8.0 Hz, *J*_2_ = 1.6 Hz, 1H, CH, H6), 7.86 (d, *J* = 1.6 Hz, 1H, CH, H8), 7.93 (d, *J* = 8.0 Hz, 1H, CH, H5). ^13^C-NMR (CDCl_3_) δ 13.0, 18.7, 24.8, 29.9, 32.3, 53.6, 58.1, 60.0, 62.7, 119.5, 126.7, 126.8, 132.0, 133.5, 134.8, 138.3, 148.1, 148.2, 175.7, 179.8. Elemental analysis calcd for C_20_H_22_N_2_O_4_: C 67.78; H 6.26; N 7.90; found: C 67.85; H 6.31; N 7.95.

*2-{7-[2-(3,3-Dimethyloxiranyl)-ethyl]-3-methyl-4,9-dioxo-4,9-dihydro-benzo-[f]indazol-1-yl}-ethyl acetate* (**3c**): This compound was prepared following the general procedure from 1-(2-acetoxyethyl)-3-methyl-7-(4-methylpent-3-enyl)-1*H-*benzo[*f*]indazole-4,9-dione **2c**. Yellow solid purified with 2:1 hexane/ethyl acetate, 64% yield, m.p. 104–106 °C; IR (NaCl, ν/cm^−1^) 1744, 1669 (C=O). ^1^H-NMR (CDCl_3_) δ 1.50 (s, 3H, CH_3_, H16), 1.63 (s, 3H, CH_3_, H15), 1.98 (s, 3H, CH_3_, R), 1.89–1.94 (m, 2 H, CH_2_, H12), 2.61 (s, 3H, CH_3_, H10), 2.77 (t, *J* = 6.2Hz, 1H, CH, H13), 2.92 (t, *J* = 6.5 Hz, 2H, CH_2_, H11), 4.53 (t, *J* = 5.3 Hz, 2 H, CH_2_N, R), 4.88 (t, *J* = 5.3 Hz, 2 H, CH_2_O, R), 7.59 (dd, *J*_1_ = 7.9 Hz, *J*_2_ = 1.6 Hz, 1H, CH, H6), 8.01 (d, *J* = 1.6 Hz, 1H, CH, H8), 8.14 (d, *J* = 7.9 Hz, 1H, CH, H5). ^13^C-NMR (CDCl_3_) δ 13.1, 18.7, 20.7, 24.7, 30.2, 32.9, 50.3, 58.3, 62.3, 63.4, 120.0, 126.7, 127.3, 132.4, 133.4, 133.5, 134.5, 147.7, 149.6, 170.5, 176.3, 180.1. Elemental analysis calcd for C_22_H_24_N_2_O_5_: C 66.65; H 6.10; N 7.07; found: C 66.60; H 6.15; N 7.14.

#### 3.1.3. General Procedure for the Preparation of *N*-Substituted 3-(3-Methyl-4,9-dioxo-4,9-dihydro-1*H*-benzo[*f*]indazol-7-yl)-propanal **4a**–**c**

These compounds were synthesized by the degradative oxidation of epoxides **3a**–**c** (0.31 mmol) dissolved in THF (10 mL) with H_5_IO_6_ (0.140 g, 0.61 mmol) in H_2_O (3 mL) stirred 1 h at r.t. After diluting with diethyl ether (20 mL), the organic phase was washed with a 5% aqueous solution of Na_2_S_2_O_7_ (4 × 10 mL) and 5% Na_2_CO_3_ (10 mL). The products were purified by column chromatography with *n*-hexane/ethyl acetate as the eluent.

*3-(3-Methyl-4,9-dioxo-4,9-dihydro-1H-benzo[f]indazol-7-yl)-propanal* (**4a**): This compound was prepared following the general procedure from epoxide **3a**. Yellow solid purified with 1:4 hexane/ethyl acetate, 72% yield, m.p. 248–250 °C; IR (NaCl, ν/cm^−1^) 3198 (NH), 1720, 1666 (C=O). ^1^H-NMR (DMSO-*d*_6_) δ 2.56 (s, 3H, CH_3,_ H10), 2.88 (t, *J* = 7.2 Hz, 2H, CH_2_, H12), 3.00 (t, *J* = 7.2 Hz, 2H, CH_2_, H11), 7.69 (dd, *J*_1_ = 7.8 Hz, *J*_2_ = 1.6 Hz, 1 H, CH, H6), 7.98 (d, *J* = 1.6 Hz, 1H, CH, H8), 8.10 (d, *J* = 7.8 Hz, 1H, CH, H5), 9.71 (s, 1H, H13), 13.7 (s, 1H, NH, R). ^13^C-NMR (DMSO-*d*_6_) δ 11.6, 28.6, 45.1, 116.6, 128.2, 129.9, 131.9, 133.6, 134.1, 142.3, 142.6, 145.1, 171.9, 180.5, 201.1. Elemental analysis calcd for C_15_H_12_N_2_O_3_: C 67.16; H 4.51; N 10.44; found: C 68.03; H 4.30; N 10.53.

*3-[1-(2-Hydroxyethyl)-3-methyl-4,9-dioxo-4,9-dihydro-1H-benzo[f]indazol-7-yl)-propanal* (**4b**): This compound was prepared following the general procedure from epoxide **3b**. Yellow orange solid purified with 1:1 hexane/ethyl acetate, 96% yield, m.p. 141–143 °C; IR (NaCl, ν/cm^−1^) 3382 (OH), 1722, 1662 (C=O). ^1^H-NMR (CDCl_3_) δ 2.52 (s, 3H, CH_3_ H10), 2.55 (t, *J* = 7.4 Hz, 2H, CH_2_, H12), 3.07 (t, *J* = 7.4 Hz, 2H, CH_2_, H11), 3.84 (t, *J* = 5.7 Hz, 2H, CH_2_N, R), 4.64 (t, *J* = 5.7 Hz, 2H, CH_2_O, R), 4.96 (s, 1H, OH, R), 7.75 (dd, *J*_1_ = 7.8 Hz, *J*_2_ = 1.6 Hz, 1H, CH, H6), 7.98 (d, *J* = 1.6 Hz, 1H, CH, H8), 8.01 (d, *J* = 7.8 Hz, 1H, CH, H5), 9.71 (s, 1H, H13). ^13^C-NMR (CDCl_3_) δ 13.1, 27.7, 44.4, 53.6, 60.0, 119.5, 126.7, 126.8, 132.1, 133.6, 134.8, 138.4, 146.8, 148.1, 175.4, 179.8, 202.4. Elemental analysis calcd for C_17_H_16_N_2_O_4_: C 65.38; H 5.16; N 8.97; found: C 65.41; H 5.19; N 9.00.

*2-[3-Methyl-4,9-dioxo-7(3-oxopropyl)-4,9-dihydro-benzo[f]indazol-1-yl]-ethyl acetate* (**4c**): This compound was prepared from epoxide **3c** following the general procedure. Yellow solid purified with 1:1 hexane/ethyl acetate, 98% yield, m.p. 90–91 °C; IR (NaCl, ν/cm^−1^) 1732, 1718, 1666 (C=O). ^1^H-NMR (CDCl_3_) δ 1.97 (s, 3H, CH_3_, R), 2.61 (s, 3H, CH_3_, H10), 2.90 (t, *J* = 7.4 Hz, 2H, CH_2_, H12), 3.10 (t, *J* = 7.4 Hz, 2H, CH_2_, H11), 4.53 (t, *J* = 5.2 Hz, 2H, CH_2_N, R), 4.87 (t, *J* = 5.2 Hz, 2H, CH_2_O, R), 7.59 (dd, *J*_1_ = 7.9 Hz, *J*_2_ = 1.7 Hz, 1H, CH, H6), 7.99 (d, *J* = 7.9 Hz, 1H, CH, H8), 8.09 (d, *J* = 1.7 Hz, 1H, CH, H5), 9.80 (s, 1H, H13). ^13^C-NMR (CDCl_3_) δ 13.1, 20.7, 27.9, 44.4, 50.2, 62.2, 120.2, 126.5, 127.4, 132.6, 133.6, 133.8, 134.5, 146.7, 149.6, 175.6, 176.2, 180.0, 200.2. Elemental analysis calcd for C_19_H_18_N_2_O_5_: C 64.40; H 5.11; N 7.90; found: C 64.35; H 5.09; N 7.94.

#### 3.1.4. General Procedure for the Preparation of *N*-Substituted 3-(3-Methyl-4,9-dioxo-4,9-dihydro-1*H*-benzo[*f*]indazol-7-yl)-propanoic Acids **5a**–**c**

These compounds were prepared via the oxidation of aldehydes **4a**–**c** (0.33 mmol) in *t*-BuOH (9 mL) with NaClO_2_ (0.5 mL aqueous solution 25%), NaH_2_PO_4_ (0.4 mL aqueous solution 5%) and catalytic 2-methyl-2-butene (0.2 mL) at r.t. for 72 h. After acid work-up with 2 M HCl and extraction with ethyl acetate (3 × 10 mL) and CH_2_Cl_2_ (10 mL), the products were purified by column chromatography with hexane/ethyl acetate 2:1 as the eluent.

*3-(3-Methyl-4,9-dioxo-4,9-dihydro-1H-benzo[f]indazol-7-yl)-propanoic acid* (**5a**): Following the general procedure, this compound was obtained from aldehyde **4a**. White solid, 98% yield, m.p. 288–290 °C; IR (NaCl, ν/cm^−1^) 3410 (OH), 3204 (NH), 1704, 1667 (C=O). ^1^H-NMR (DMSO-*d*_6_) δ 2.52 (s, 3H, CH_3_, H10), 2.62 (t, *J* = 7.4 Hz, 2H, CH_2_, H12), 2.98 (t, *J* = 7.4 Hz, 2H, CH_2_, H11), 7.71 (dd, *J*_1_ = 8.2 Hz, *J*_2_ = 1.6 Hz, 1H, CH, H6), 7.93 (d, *J* = 1.6 Hz, 1H, CH, H8), 7.99 (d, *J* = 8.2 Hz, 1H, CH, H5), 12.23 (s, broad, 1H, H13), 14.20 (s, 1H, NH, R). ^13^C-NMR (DMSO-*d*_6_) δ 13.9, 30.1, 34.3, 114.1, 117.9, 126.5, 134.0, 134.2, 147.3, 173.4, 179.6. Elemental analysis calcd for C_15_H_12_N_2_O_4_: C 63.37; H 4.25; N 9.85; found: C 63.41; H 4.30; N 9.65.

*3-[1-(2-Hydroxyethyl)3-methyl-4,9-dioxo-4,9-dihydro-1H-benzo[f]indazol-7-yl]-propanoic acid* (**5b**): Following the general procedure, this compound was obtained from aldehyde **4b**. Yellow solid, 64% yield, m.p. 218–219 °C; IR (NaCl, ν/cm^−1^) 3331 (OH), 1700, 1664 (C=O). ^1^H-NMR (DMSO-*d*_6_) δ 2.50 (s, 3H, CH_3_, H10), 2.63 (t, *J* = 7.4 Hz, 2H, CH_2_, H12), 2.98 (t, *J* = 7.4 Hz, 2H, CH_2_, H11), 3.77 (t, *J* = 5.5 Hz, 2H, CH_2_N, R), 4.58 (t, *J* = 5.5 Hz, 2H, CH_2_O, R), 4.90 (s, 1H, OH, R), 7.71 (dd, *J*_1_ = 7.9 Hz, *J*_2_ = 1.5 Hz, 1H, CH, H6), 7.90 (d, *J* = 1.5 Hz, 1H, CH, H8), 8.00 (d, *J* = 1.5 Hz, 1H, CH, H5), 12.30 (s, broad, 1H, H13). ^13^C-NMR (DMSO-*d*_6_) δ 13.1, 30.5, 34.7, 53.6, 60.0, 119.5, 126.7, 126.8, 132.1, 133.6, 134.8, 147.8, 148.1, 173.5, 175.8, 179.9. Elemental analysis calcd for C_17_H_16_N_2_O_5_: C 62.19; H 4.91; N 8.53; found: C 62.14; H 4.86; N 8.48.

*3-[1-(2-Acetoxyethyl)-3-methyl-4,9-dioxo-4,9-dihydro-1H-benzo[f]indazol-7-yl]-propanoic acid* (**5c**): Following the general procedure, this compound was obtained from aldehyde **4c**. Grey solid, 48% yield, m.p. 186–187 °C; IR (NaCl, ν/cm^−1^) 3400 (OH), 1742, 1705, 1670, 1664 (C=O). ^1^H-NMR (DMSO-*d*_6_) δ 1.90 (s, 3H, CH_3_, R), 2.50 (s, 3H, CH_3_, H10), 2.64, (t, *J* = 7.4 Hz, 2H, CH_2_, H12), 3.00 (t, *J* = 7.4 Hz, 2H, CH_2_, H11), 4.45 (t, *J* = 5.2 Hz, 2H, CH_2_N, R), 4.79 (t, *J* = 5.2 Hz, 2H, CH_2_O, R), 7.74 (dd, *J*_1_ = 7.9 Hz, *J*_2_ = 1.4 Hz, 1H, H6), 7.97 (d, *J* = 1.4 Hz, 1H, CH, H8), 8.00 (d, *J* = 7.9 Hz, 1H, CH, H5), 12.05 (s, broad, 1H, H13). ^13^C-NMR (DMSO-*d*_6_) δ 12.7, 20.4, 30.1, 34.3, 49.8, 61.8, 119.3, 126.3, 126.5, 131.7, 133.1, 138.1, 148.0, 170.0, 173.4, 174.5, 179.5. Elemental analysis calcd for C_19_H_18_N_2_O_6_: C 61.62; H 4.96; N 7.50; Found: C 61.55; H 4.90; N 7.60.

#### 3.1.5. General Procedure for the Preparation of [3-(3-Methyl-4,9-dioxo-4,9-dhydro-1*H*-benzo[*f*]indazol-7-yl)propanoylamino]-methyl Ester **6a**–**l**

A solution containing 0.37 mmol of carboxylic acids **5a**–**c**, 0.041 g (0.407 mmol, 56 μL) of triethylamine and 0.044 g (0.407 mmol, 38 μL) of ethyl chloroformate in 12 mL of dry THF was stirred for 20 min at 0 °C. After the addition of 0.407 mmol of the protected l-amino acid (Gly, Ala, Phe, and Glu), the mixture was stirred 16 h at r.t. After filtration over Celite-545 and evaporation of the solvent, the residue was dissolved in 70 mL of ethyl acetate. The organic solution was extracted with 40 mL of a 5% NaHCO_3_ solution and water (40 mL). After drying with Na_2_SO_4_, the solvent was removed under reduced pressure, and the crude product was purified by column chromatography with chloroform/acetone 7:3 as the eluent.

*[3-(3-Methyl-4,9-dioxo-4,9-dihydro-1H-benzo[f]indazol-7-yl)propanoylamino]-methyl acetate* (**6a**): This compound was prepared following the general procedure from carboxylic acid **5a** and glycine methyl ester hydrochloride. White solid, 80% yield, m.p. 213–215 °C; IR (NaCl, ν/cm^−1^) 3219 (NH), 1750, 1668, 1640 (C=O). ^1^H-NMR (DMSO-*d*_6_) δ 2.53 (t, *J* = 7.6 Hz, 2H, CH_2_, H12), 2.57 (s, 3H, CH_3_, H10), 2.99 (t, *J* = 7.6 Hz, 2H, CH_2_, H11), 3.59 (s, 3H, CH_3_O), 3.81 (d, *J* = 5.8 Hz, 2H, CH_2_), 7.70 (dd, *J*_1_ = 7.9 Hz, *J*_2_ = 1.5 Hz, 1H, CH, H6), 7.96 (d, *J* = 1.5 Hz, 1H, CH, H8), 7.99 (d, *J* = 7.9 Hz, 1H, CH, H5), 8.35 (d, *J* = 5.0 Hz, 1H, NH), 14.26 (s, 1H, NH, R). ^13^C-NMR (DMSO-*d*_6_) δ 11.0, 30.7, 35.7, 40.5, 51.6, 114.1, 117.8, 126.4, 126.5, 132.8, 134.0, 147.5, 170.8, 171.5, 179.6. Elemental analysis calcd for C_18_H_17_N_3_O_5_: C 60.84; H 4.82; N 11.82; found: C 60.85; H 4.87; N 11.90.

*2-[3-(3-Methyl-4,9-dioxo-4,9-dihydro-1H-benzo[f]indazol-7-yl)propanoylamino]-methyl propanoate* (**6b**): This compound was prepared following the general procedure from carboxylic acid **5a** and l-alanine methyl ester hydrochloride. Yellow solid, 49% yield, m.p. 246–248 °C; IR (NaCl, ν/cm^−1^) 3221 (NH), 1743, 1667, 1645 (C=O). ^1^H-NMR (DMSO-*d*_6_) δ 1.21 (d, *J* = 7.3 Hz, 3H, CH_3_, R_1_), 2.49 (t, *J* = 7.4 Hz, 2H, CH_2_, H12), 2.57 (s, 3H, CH_3_, H10), 2.95 (t, *J* = 7.4 Hz, 2H, CH_2_, H11), 3.57 (s, 3H, CH_3_O), 4.20 (q, *J* = 7.3 Hz, 1H, CH), 7.69 (dd, *J*_1_ = 8.0 Hz; *J*_2_ = 1.6 Hz, 1H, CH, H6), 7.96 (d, *J* = 1.6 Hz, 1H, CH, H8), 8.01 (d, *J* = 8.0 Hz, 1H, CH, H5), 8.34 (d, *J* = 7.0 Hz, 1H, NH), 14.10 (s, 1H, NH, R). ^13^C-NMR (DMSO-*d*_6_) δ 10.8, 17.0, 30.7, 42.7, 51.7, 117.8, 126.3, 126.4, 126.6, 126.7, 134.0, 134.2, 147.3, 170.8, 173.0, 179.0. Elemental analysis calcd for C_19_H_19_N_3_O_5_: C 61.78; H 5.18; N 11.38; found: C 61.85; H 5.20; N 11.43.

*2-[3-(3-Methyl-4,9-dioxo-4,9-dihydro-1H-benzo[f]indazol-7-yl)propanoylamino]-methyl-3-phenyl propanoate* (**6c**): This compound was prepared following the general procedure from carboxylic acid **5a** and l-phenylalanine methyl ester hydrochloride. Brown solid, 98% yield, m.p. 215–217 °C; IR (NaCl, ν/cm^−1^) 3219 (NH), 1774, 1691, 1667 (C=O). ^1^H-NMR (CDCl_3_) δ 2.66 (t, *J* = 7.9 Hz, 2H, CH_2_, H12), 2.85 (s, 3H, CH_3_, H10), 3.06–3.12 (m, 4H, 2 CH_2_, H11, R_1_), 3.75 (s, 3H, CH_3_O), 4.13 (q, *J* = 7.2 Hz, 1H, CH), 7.17–7.60 (m, 8H, CH, aromatic), 8.11 (s, broad, 1H, NH), 13.96 (s, 1H, NH, R). ^13^C-NMR (CDCl_3_) δ 14.1, 26.5, 37.5, 40.9, 51.9, 114.5, 126.2, 126.8, 127.1, 128.5, 129.2, 133.0, 137.1, 171.0 173.0, 175.3. Elemental analysis calcd for C_25_H_23_N_3_O_5_: C 67.41; H 5.20; N 9.40; found: C 67.35; H 5.25; N 9.60.

*2-[3-(3-Methyl-4,9-dioxo-4,9-dihydro-1H-benzo[f]indazol-7-yl)propanoylamino]-dimethyl pentane-dioate* (**6d**): This compound was prepared following the general procedure from carboxylic acid **5a** and l-glutamic acid dimethyl ester hydrochloride. White solid, 38% yield, m.p. 222–224 °C; IR (NaCl, ν/cm^−1^) 3226 (NH), 1746, 1691, 1669 (C=O). ^1^H-NMR (DMSO-*d*_6_) δ 1.70–1.90 (m, 2H, CH_2_, R_1_), 2.00–2.35 (m, 2H, CH_2_, R_1_), 2.52 (t, *J* = 7.3 Hz, 2H, CH_2_, H12), 2.57 (s, 3H, CH_3_, H10), 2.98 (t, *J* = 7.3 Hz, 2H, CH_2_, H11), 3.46 (s, 3H, CH_3_O), 3.57 (s, 3H, CH_3_O), 4.10–4.30 (m, 1H, CH), 7.68 (dd, *J*_1_ = 7.9 Hz, *J*_2_ = 1.5 Hz, 1H, CH, H6), 7.92 (d, *J* = 1.5 Hz, 1H, CH, H8), 7.99 (d, *J* = 7.9 Hz, 1H, CH, H5), 8.29 (d, *J* = 7.7 Hz, NH), 14.24 (s, 1H, NH, R). ^13^C-NMR (DMSO-*d*_6_) δ 11.0, 26.0, 29.3, 30.8, 35.7, 50.8, 51.2, 51.8, 114.1, 117.8, 126.4, 126.5, 132.7, 133.5, 133.9, 134.2, 147.4, 171.1, 172.0, 172.4, 179.6. Elemental analysis calcd for C_22_H_23_N_3_O_7_: C 59.85; H 5.25; N 9.52; found: C 60.01; H 5.56; N 9.63.

{*3-[1-(2-Hydroxyethyl)-3-methyl-4,9-dioxo-4,9-dihydro-1H-benzo[f]indazol-7-yl]-propanoylamino}-methyl acetate* (**6e**): This compound was prepared following the general procedure from carboxylic acid **5b** and glycine methyl ester hydrochloride. White solid, 80% yield, m.p. 188–190 °C; IR (NaCl, ν/cm^−1^) 3325 (broad, NH, OH), 1762, 1742, 1671, 1651 (C=O). ^1^H-NMR (DMSO-*d*_6_) δ 2.50 (s, 3H, CH_3_, H10), 2.53 (t, *J*= 7.5 Hz, 2H, CH_2_, H12), 2.99 (t, *J* = 7.5 Hz, 2H, CH_2_, H11), 3.60 (s, 3H, CH_3_O), 3.77 (d, *J*= 5.6 Hz, 2H, CH_2_), 3.81 (t, *J* = 5.6 Hz, 2H, CH_2_, CH_2_N, R), 4.61 (t, *J* = 5.6 Hz, 2H, CH_2_O, R), 4.92 (s, 1H, OH, R), 7.71 (dd, *J*_1_ = 8.0 Hz, *J*_2_ = 1.6 Hz, 1H, CH, H6), 7.93 (d, *J* = 1.6 Hz, 1H, CH, H8), 7.97 (*J* = 8.0 Hz, 1H, CH, H5), 8.38 (t, *J* = 5.6 Hz, 1H, NH). ^13^C-NMR (DMSO-*d*_6_) δ 12.8, 30.4, 30.7, 35.7, 40.5, 51.6, 53.3, 59.6, 119.2, 124.9, 126.4, 126.5, 131.7 133.3, 134.5, 138.1, 147.8, 170.3, 171.5, 175.5, 179.6. Elemental analysis calcd for C_20_H_21_N_3_O_6_: C 60.15; H 5.30; N 10.52; found: C 60.19; H 5.34; N 10.53.

2-{*3-[1-(2-Hydroxyethyl)-3-methyl-4,9-dioxo-4,9-dihydro-1H-benzo[f]indazol-7-yl]-propanoylamino}-methyl propanoate* (**6f**): This compound was prepared following the general procedure from carboxylic acid **5b** and l-alanine methyl ester hydrochloride. Yellow solid, 49% yield, m.p. 181–182 °C; IR (NaCl, ν/cm^−1^) 3317 (broad, NH, OH), 1729, 1663, 1647 (C=O). ^1^H-NMR (DMSO-*d*_6_) δ 1.22 (d, *J* = 7.2 Hz, 3H, CH_3_, R_1_); 2.50 (s, 3H, CH_3_, H10), 2.52 (t, *J* = 7.2 Hz, 2H, CH_2_, H12), 2.98 (t, *J* = 7.2 Hz, 2H, CH_2_, H11), 3.59 (s, 3H, CH_3_O), 3.79 (t, *J* = 5.6 Hz, 2H, CH_2_N, R), 4.24–4.27 (m, 1H, CH), 4.61 (t, *J* = 5.6 Hz, 2H, CH_2_O, R), 4.92 (s, 1H, OH), 7.70 (dd, *J_1_* = 7.8, *J*_2_ = 1.5 Hz, 1H, CH, H6), 7.93 (d, *J*= 1.5 Hz, 1H, CH, H8), 7.97 (d, *J* = 7.8 Hz, 1H, CH, H5), 8.35 (d, *J* = 7.2 Hz, 1H, NH). ^13^C-NMR (DMSO-*d*_6_) δ 12.8, 17.0, 30.7, 35.7, 47.4, 51.7, 53.2, 59.6, 119.1, 126.4, 131.7, 133.2, 134.5, 138.1, 147.7, 170.8, 173.1, 175.5, 179.6. Elemental analysis calcd for C_21_H_23_N_3_O_6_: C 61.01; H 5.61; N 10.16; found: C 61.05; H 5.66; N 10.21.

*2*-{*3-[1-(2-Hydroxyethyl)-3-methyl-4,9-dioxo-4,9-dihydro-1H-benzo[f]indazol-7-yl]-propanoylamino}-methyl-3-phenylpropanoate* (**6g**): This compound was prepared following the general procedure from carboxylic acid **5b** and l-phenylalanine methyl ester hydrochloride. Brown solid, 98% yield, m.p. 154–156 °C. IR (NaCl, ν/cm^−1^) 3309 (broad, NH, OH), 1743, 1659, 1644 (C=O). ^1^H-NMR (DMSO-*d*_6_) δ 2.44 (t, *J* = 7.4 Hz, 2H, CH_2_, H12), 2.48 (s, 3H, CH_3_, H10), 2.75–3.00 (m, 4H, CH_2_, CH_2_, H11, R_1_), 3.56 (s, 3H, CH_3_O); 3.77 (t, *J* = 5.6 Hz, 2H, CH_2_N, R), 4.43–4.45 (m, 1H, CH) 4.61 (t, *J* = 5.6 Hz, 2H, CH_2_O, R), 4.90 (s,1H, OH), 7.12–7.22 (m, 5H, CH, aromatic, R_1_), 7.60 (dd, *J*_1_ = 7.9 Hz, *J*_2_ = 1.6 Hz, 1H, CH, H6), 7.80 (d, *J* = 1.6 Hz, 1H, CH, H8), 7.94 (d, *J* = 7.9 Hz, 1H, CH, H5), 8.39 (d, *J* = 7.8 Hz, 1H, NH). ^13^C-NMR (DMSO-*d*_6_) δ 12.8, 30.7, 35.7, 36.7, 51.8, 53.3, 53.4, 59.6, 119.2, 126.4, 128.1, 128.9, 131.7, 133.2, 134.4, 137.1, 138.1, 147.7, 147.8, 171.0, 172.0, 175.5, 179.6. Elemental analysis calcd for C_27_H_27_N_3_O_6_: C 66.25; H 5.56; N 8.58; found: C 66.21; H 5.50; N 7.92.

*2-{3-[1-(2-Hydroxyethyl)-3-methyl-4,9-dioxo-4,9-dihydro-1H-benzo[f]indazol-7-yl)propanoylamino]-dimethyl pentanedioate* (**6h**): This compound was prepared following the general procedure from carboxylic acid **5b** and l-glutamic acid dimethyl ester hydrochloride. Yellow solid, 44% yield, m.p. 160–162 °C. IR (NaCl, ν/cm^−1^) 3306 (broad, NH, OH), 1738, 1675, 1644 (C=O). ^1^H-NMR (DMSO-*d*_6_) δ 1.60–2.00 (m, 2H, CH_2_, R_1_), 2.12–2.19 (m, 2H, CH_2_, R_1_), 2.49 (s, 3H, CH_3_, H10), 2.52 (t, *J* = 7.6 Hz, 2H, CH_2_, H12), 2.96 (t, *J* = 7.6 Hz, 2H, CH_2_, H11), 3.47 (s, 3H, CH_3_O), 3.58 (s, 3H, CH_3_O), 3.78 (t, *J* = 5.7 Hz, 2H, CH_2_N, R), 4.23–4.25 (m, 1H, CH), 4.60 (t, *J* = 5.7 Hz, 2H, CH_2_O, R), 4.91(s,1H, OH), 7.68 (dd, *J*_1_ = 7.1 Hz, *J*_2_ = 1.6 Hz, 1H, CH, H6), 7.92 (d, *J* = 1.6 Hz, 1H, CH, H8), 7.97 (d, *J* = 7.1 Hz, 1H, CH, H5), 8.28 (d, *J* = 7.7 Hz, 1H, NH). ^13^C-NMR (DMSO-*d*_6_) δ 12.8, 26.0, 29.4, 30.8, 35.7, 50.9, 51.2, 51.8, 53.2, 59.6, 119.1, 126.4, 131.7, 133.2, 134.5, 138.1, 147.6, 147.7, 171.1, 172.0, 172.4, 175.5, 179.5. Elemental analysis calcd for C_24_H_27_N_3_O_8_: C 59.37; H 5.61; N 8.66; found: C 59.42; H 5.70; N 8.71.

{*3-[1-(2-Acetoxyethyl)-3-methyl-4,9-dioxo-4,9-dihydro-1H-benzo[f]indazol-7-yl]-propanoylamino}-methyl acetate* (**6i**): This compound was prepared following the general procedure from carboxylic acid **5c** and glycine methyl ester hydrochloride. Yellow oil, 41% yield. IR (NaCl, ν/cm^−1^) 3330 (broad, NH), 1742, 1670, 1664, 1658 (C=O). ^1^H-NMR (CDCl_3_) δ 1.98 (s, 3H, CH_3_, R), 2.61 (s, 3H, CH_3_, H10), 2.65 (t, *J* = 7.4 Hz, 2H, CH_2_, H12), 3.14 (t, *J* = 7.4 Hz, 2H, CH_2_, H11), 3.75 (s, 3H, CH_3_O), 4.01 (d, *J* = 5.0 Hz, 2H, CH_2_), 4.53 (t, *J* = 5.2 Hz, 2H, CH_2_N, R), 4.87 (t, *J* = 5.2 Hz, 2H, CH_2_O, R), 6.06 (d, *J* = 5.0 Hz, 1H, NH), 7.62 (dd, *J*_1_ = 7.9 Hz, *J*_2_ = 1.6 Hz, 1H, CH, H6), 8.00 (d, *J* = 1.6 Hz, 1H, CH, H8), 8.12 (d, *J* = 7.9 Hz, 1H, CH, H5). ^13^C-NMR (CDCl_3_) δ 13.5, 21.1, 30.1, 31.6, 31.7, 37.1, 37.2, 41.7, 50.7, 52.8, 62.7, 120.7, 127.0, 127.8, 133.0, 134.1, 135.0, 138.7, 147.5, 148.8, 150.0, 170.3, 171.0, 171.1, 176.7, 180.5. Elemental analysis calcd for C_22_H_23_N_3_O_7_: C 59.85; H 5.25; N 9.52; found: C 59.91; H 5.32; N 9.85.

2-{*3-[1-(2-Acetoxyethy)-3-methyl-4,9-dioxo-4,9-dihydro-1H-benzo[f]indazol-7-yl]-propanoylamino}-methyl propanoate* (**6j**): This compound was prepared following the general procedure from carboxylic acid **5c** and l-alanine methyl ester hydrochloride. Amber solid, 56% yield, m.p. 130–132 °C. IR (NaCl, ν/cm^−1^) 3312 (broad, NH), 1744, 1670, 1654, 1648 (C=O). ^1^H-NMR (CDCl_3_) δ 1.31 (d, *J* = 7.1 Hz, 3H, CH_3_, R_1_), 1.91 (s, 3H, CH_3_, R), 2.50 (s, 3H, CH_3_, H10), 2.52 (t, *J* = 7.7 Hz, 2H, CH_2_, H12), 3.06 (t, *J* = 7.7 Hz, 2H, CH_2_, H11), 3.67 (s, 3H, CH_3_O,), 4.45 (t, *J* = 5.2 Hz, 2H, CH_2_N, R), 4.49 (q, *J* = 7.1 Hz, 1H, CH), 4.80 (t, *J* = 5.2 Hz, 2H, CH_2_O, R), 5.99 (d, *J* = 7.1 Hz, 1H, NH), 7.54 (dd, *J*_1_ = 7.9 Hz, *J*_2_ = 1.5 Hz, 1H, CH, H6), 7.93 (d, *J* = 1.5 Hz, 1H, CH, H8), 8.06 (d, *J* = 7.9 Hz, 1H, CH, H5). ^13^C-NMR (CDCl_3_) δ 13.5, 18.9, 21.1, 30.1, 31.6, 37.4, 48.4, 49.0, 50.7, 52.9, 125.5, 127.0, 127.8, 130.3, 132.2, 134.0, 135.0, 147.5, 150.0, 171.0, 171.5, 173.9, 176.7, 180.9. Elemental analysis calcd for C_23_H_25_N_3_O_7_: C 60.65; H 5.53; N 9.23; found: C 60.50; H 5.60; N 9.30.

*2*-{*3-[1-(2-Acetoxyethyl)-3-methyl-4,9-dioxo-4,9-dihydro-1H-benzo[f]indazol-7-yl]-propanoylamino}-methyl 3-phenylpropanoate* (**6k**): This compound was prepared following the general procedure from carboxylic acid **5c** and l-phenylalanine methyl ester hydrochloride. Yellow oil, 69% yield. IR (NaCl, ν/cm^−1^) 3302 (broad, NH), 1744, 1668, 1648 (C=O). ^1^H-NMR (CDCl_3_) δ 1.97 (s, 3H, CH_3_, R), 2.57 (t, *J* = 7.5 Hz, 2H, CH_2_, H12), 2.62 (s, 3H, CH_3_, H10), 3.07–3.10 (m, 4H, CH_2_, CH_2_, H11, R_1_), 3.72 (s, 3H, CH_3_O), 4.52 (t, *J* = 5.3 Hz, 2H, CH_2_N, R), 4.86–4.88 (m, 3H, CH, CH_2_O, R), 5.88 (d, *J* = 7.6 Hz, 1H, NH), 7.23–7.27 (m, 5H, aromatic CH, R_1_), 7.58 (dd, *J*_1_ = 8.0 Hz, *J*_2_ = 1.6 Hz, 1H, CH, H6), 7.99 (d, *J* = 1.6 Hz, 1H, CH, H8), 8.12 (d, *J* = 8.0 Hz, 1H, CH, H5). ^13^C-NMR (CDCl_3_) δ 13.5, 21.1, 31.5, 37.4, 38.2, 50.7, 52.8, 53.4, 120.2, 127.0, 127.6, 127.8, 129.0, 129.6, 134.0, 135.0, 136.0, 147.4, 150.0, 171.0, 171.1, 172.3, 180.2. Elemental analysis calcd for C_29_H_29_N_3_O_7_: C 65.53; H 5.50; N 7.90; found: C 65.48; H 5.60; N 7.96.

*2-{3-[1-(2-Acetoxyethyl)-3-methyl-4,9-dioxo-4,9-dihydro-1H-benzo[f]indazol-7-yl)propanoylamino]-dimethyl pentanedioate* (**6l**): This compound was prepared following the general procedure from carboxylic acid **5c** and l-glutamic acid dimethyl ester hydrochloride. Yellow oil, 48% yield. IR (NaCl, ν/cm^−1^) 3330 (broad, NH), 1740, 1670, 1644 (C=O). ^1^H-NMR (CDCl_3_) δ 1.99 (s, 3H, CH_3_, R), 2.10–2.40 (m, 4H, CH_2_, CH_2_, R_1_), 2.60 (s, 3H, CH_3_, H10), 2.62 (t, *J* = 7.8 Hz, 2H, CH_2_, H12), 3.12 (t, *J* = 7.8 Hz, 2H, CH_2_, H11), 3.63 (s, 3H, CH_3_O), 3.76 (s, 3H, CH_3_O), 4.53 (t, *J* = 5.2 Hz, 2H, CH_2_N, R), 4.60 (d, *J* = 5.0 Hz, 1H, CH), 4.80 (t, *J* = 5.2 Hz, 2H, CH_2_O, R), 6.26 (d, *J* = 7.4 Hz, 1H, NH), 7.62 (dd, *J*_1_ = 7.9 Hz, *J*_2_ = 1.7 Hz, 1H, CH, H6), 8.01 (d, *J* = 1.7 Hz, 1H, CH, H8), 8.12 (d, *J* = 7.9 Hz, 1H, CH, H5). ^13^C-NMR (CDCl_3_) δ 13.5, 21.1, 27.6, 30.3, 31.6, 37.4, 50.7, 52.1, 52.3, 53.0, 122.0, 127.0, 127.8, 134.1, 135.0, 138.7, 147.4, 150.1, 171.0, 171.5, 172.6, 173.7, 175.0, 180.1. Elemental analysis calcd for C_26_H_29_N_3_O_9_: C 59.20; H 5.54; N 7.97; found: C 59.13; H 5.72; N 8.01.

### 3.2. Computational Details

DFT calculations [30,31,32,33] were conducted using the Amsterdam Density Functional (ADF) program [34]. The Vosko-Wilk-Nusair parametrization [35] was used to treat electron correlation within the local density approximation (LDA). The numerical integration procedure applied for the calculation was developed by teVelde [33]. The standard ADF TZ2P basis set was used for all atoms. The frozen core approximation was used to treat core electrons at the following levels: C, 1s; N, 1s; and O, 1s [33]. Full geometry optimizations were performed on each complex using the analytical gradient method implemented by Verluis and Ziegler [36]. The geometries for all the model compounds discussed in the text were fully optimized and checked via analytical frequency calculations as either true minima (no imaginary values).

### 3.3. Antiproliferative Assay

KATO-III (human gastric cancer cell line) and MCF-7 (human breast adenocarcinoma cell line) cells were obtained from the American Type Culture Collection (ATCC). KATO-III and MCF-7 cells (2 × 10^3^) were grown in DMEM supplemented with 10% FBS and 1% penicillin/streptomycin. Cells were subcultured into fresh medium (100-mm-diameter plate dish) until a density of approximately 80% was obtained. Briefly, 2 × 10^3^ cells were seeded in 96-well culture plates. After 24 h of incubation at 37 °C in a humidified 5% CO_2_ atmosphere, different concentrations (10^−9^ to 10^−3^ M) of 1*H*-benzo[*f*]indazole-4,9-dione-based derivatives were added. After 72 h of incubation, 20 μL of MTS (Promega, Madison, WI, USA) was added, and the wells were incubated for an additional 2 h at 37 °C. The absorbance at 490 nm was recorded using a Varioskan Flash Multimode Reader (Thermo Scientific, Waltham, MA, USA). Each variant of the experiment was performed in triplicate. To obtain IC_50_ values for each compound, dose-response curves were constructed in both KATO-III and MCF-7 cell lines. Doxorubicin was included in all evaluation to provide a reference of antiproliferative activity.

### 3.4. Statistical Analysis

Data are expressed as means ± CI 95% (95% confidence intervals) for three independent experiments. The concentration inducing a 50% decrease of cell proliferation (IC_50_) was performed using the four-parameters logistic fit—also known as “4PL”—supported by GraphPad Prism 6 (GraphPad Software, San Diego, CA, USA). Statistical differences among means were assessed using a two-way ANOVA test followed by a Dunnett’s multiple comparison post-test. A *p* < 0.05 was taken as statistically significant.

## 4. Conclusions

In this study, we have synthesized three series of new 1*H*-benzo[*f*]indazole-4,9-dione-based derivatives containing oxiranyl, formyl, carboxylic and l- and *C*-protected *N*-aminoacidyl substituents attached to the side chain of the 1,4-naphthoquinone group in moderate to good yields. All compounds were characterized using spectroscopic techniques, namely, FT-IR, ^1^H-NMR, and ^13^C-NMR, and their data are in agreement with their proposed structures. DFT calculations provide the first insights into the reaction pathways; further investigations to clarify the entire reaction pathway are currently in progress. These families of compounds contain, in a single structure, a 1,4-quinone group fused to a pyrazolyl heterocyclic ring, substituents that are present individually in anticancer drugs such as doxorubicin, daunorubicin or in heterocyclic compounds with antitumoral properties. Moreover, they contain an amino acid group capable of orienting their transport into the cell organelles, where they could interfere with protein synthesis. Preliminary antiproliferative activity analyses showed that most of the derivatives presented some degree of activity. However, the derivatives **2**, **4** and those conjugated with l-alanine, l-phenylalanine and l-glutamic acid, and especially those belonging to Series-II and -III, presented the highest activity, as indicated by their IC_50_ values. These results suggest that 1*H*-benzo[*f*]indazole-4,9-dione-based derivatives are promising compounds for the development of anticancer drugs.

## References

[1] Foye W.O. (1995). Cancer Chemotherapeutic Agents.

[2] Hertzberg R.P., Dervan P.B. (1984). Cleavage of DNA with methidiumpropyl-EDTA-iron(II): Reaction conditions and product analyses. Biochemistry.

[3] Lee H., Suh M., Lee C. (2003). Synthesis and cytotoxicity evaluation of 2-amino- and 2-hydroxy-3-ethoxycarbonyl-*N*-substituted-benzo[*f*]indole-4,9-dione derivatives. Bioorg. Med. Chem..

[4] Brunmark A., Cadenas E. (1989). Redox and addition chemistry of quinoid compounds and its biological implications. Free Radic. Biol. Med..

[5] O’Brien P.J. (1991). Molecular mechanisms of quinone cytotoxicity. Chem Biol Interact.

[6] Montoya J., Varela-Ramírez A., Shanmugasundram M., Martínez L.E., Primm T.P., Aguilera R.J. (2005). Tandem screening of toxic compounds on GFP-labeled bacteria and cancer cells in microtiter plates. Biochem. Biophys. Res. Commun..

[7] Benites J., Valderrama J.A., Rivera F., Rojo L., Campos N., Pedro M., Nascimento M.S. (2008). Studies on quinones. Part 42: Synthesis of furylquinone and hydroquinones with antiproliferative activity against human tumor cell lines. Part 42. Bioorg. Med. Chem..

[8] Gaikwad D.D., Chapolikar A.D., Devkate C.G., Warad K.D., Tayade A.P., Pawar R.P., Domb A.J. (2015). Synthesis of indazole motifs and their medicinal importance: An overview. Eur. J. Med. Chem..

[9] Thangadurai A., Minu M., Wakode S., Agrawal S., Narasimhan B. (2012). Indazole: A medicinally important heterocyclic moiety. Med. Chem. Res..

[10] Conway G.A., Loeffler L.J., Hall I.H. (1983). Synthesis and antitumor evaluation of selected 5,6-disubstituted 1(2)*H*-indazole-4,7-diones. J. Med. Chem..

[11] Zhao C., Wang R., Li G., Xue X., Sun C., Qu X., Li W. (2013). Synthesis of indazole based diarylurea derivatives and their antiproliferative activity against tumor cell lines. Bioorg. Med. Chem. Lett..

[12] Buchstaller H., Eggenweiler H., Sirrenberg C., Grädler U., Musil D., Hoppe E., Zimmermann A., Schwartz H., März J., Bomke J. (2012). Fragment-based discovery of hydroxy-indazole-carboxamides as novel small molecule inhibitors of Hsp90. Bioorg. Med. Chem. Lett..

[13] Shakil N.A., Singh M.K., Sathiyendiran M., Kumar J., Padaria J.C. (2013). Microwave synthesis, characterization and bio-efficacy evaluation of novel chalcone based 6-carbethoxy-2-cyclohexen-1-one and 2*H*-indazol-3-ol derivatives. Eur. J. Med. Chem..

[14] Wilhelm S.M., Adnane L., Newell P., Villanueva A., Llovet J.M., Lynch M. (2008). Preclinical overview of sorafenib, a multikinase inhibitor that targets both Raf and VEGF and PDGF receptor tyrosine kinase signaling. Mol. Cancer Ther..

[15] Flaherty K.T. (2007). Sorafenib in renal cell carcinoma. Clin. Cancer Res..

[16] Villanueva A., Llovet J.M. (2012). Second-line therapies in hepatocellular carcinoma: Emergence of resistance to sorafenib. Clin. Cancer Res..

[17] Takezawa K., Okamoto I., Yonesaka K., Hatashita E., Yamada Y., Fukuoka M., Nakagawa K. (2009). Sorafenib inhibits non-small cell lung cancer cell growth by targeting B-RAF in KRAS wild-type cells and C-RAF in KRAS mutant cells. Cancer Res..

[18] Clark J.W., Eder J.P., Ryan D., Lathia C., Lenz H. (2005). Safety and pharmacokinetics of the dual action Raf kinase and vascular endothelial growth factor receptor inhibitor, BAY 43–9006, in patients with advanced, refractory solid tumors. Clin. Cancer Res..

[19] Fieser L.F., Peters M.A. (1931). The addition of diazomethane and some of its derivatives to alpha-naphthoquinone. J. Am. Chem. Soc..

[20] Brockmann H., Reschks T. (1965). Die struktur der *N*-methyl-l-in-naphtindazolchinone-(4,9). Tetrahedron Lett..

[21] Eistert B., Pfleger K., Arackal T.J., Holzer G. (1975). Reactions of quinones and α-dicarbonyl compounds with diazoalkanes: XXII Reactions of 2,3-dichloro-*p*-benzoquinone with diazoalkanes. Chem. Ber..

[22] Baxter I., Davis B.A. (1971). Synthesis of heterocyclic quinones. Q. Rev. Chem. Soc..

[23] Molinari A., Oliva A., Arismendi M., Imbarack E., Gálvez C., Maldonado J., San Feliciano A. (2015). The synthesis of some fused pyrazolo-1,4-naphthoquinones. J. Heterocycl. Chem..

[24] Molinari A., Ojeda C., Oliva A., Miguel del Corral J.M., Castro M.A., García P.A., Cuevas C., San Feliciano A. (2009). Synthesis, characterization, and antineoplastic cytotoxicity of hybrid naphthohydroquinone-nucleic base mimic derivatives. Med. Chem. Res..

[25] Molinari A., Oliva A., Ojeda C., del Corral J.M.M., Castro M.A., Molinedo F., San Feliciano A. (2009). Synthesis and evaluation as antitumor agents of 1,4-naphthohydroquinone derivatives conjugated with amino acids and purines. Arch. Pharm. Chem. Life Sci..

[26] Wu A.A., Xu Y., Qian X. (2009). Novel naphthalimide-amino acid conjugated with flexible leucine moiety as side chain: design, synthesis and potential antitumor activity. Bioorg. Med. Chem..

[27] Wu W., Dong Y., Gao J., Gong M., Zhang X., Kong W., Li Y., Zeng Y., Si D., Wei Z. (2015). Aspartate-modified doxorubicin on its *N*-terminal increases drug accumulation in *LAT1*-overexpressing tumors. Cancer Sci..

[28] Kutyrev A.A. (1991). Nucleophilic reactions of quinones. Tetrahedron.

[29] Miguel del Corral J., Gordaliza M., Angeles Castro M., Mahiques M.M., San Feliciano A., García-Grávalos M.D. (1998). Further antineoplastic terpenylquinones and terpenylhydroquinones. Bioorg. Med. Chem..

[30] Baerends E.J., Ellis D.E., Ros P. (1973). Self-consistent molecular Hartree-Fock-Slater calculations I. The computational procedure. Chem. Phys..

[31] Baerends E.J., Ros P. (1978). Evaluation of the LCAO Hartree-Fock-Slater method: Applications to transition-metal complexes. Int. J. Quant Chem..

[32] Boerrigter P.M., Te Velde G., Baerends J.E. (1988). Three-dimensional numerical integration for electronic structure calculations. Int. J. Quant Chem..

[33] Te Velde G., Baerends E.J. (1992). Numerical integration for electronic structure calculations. J. Comput. Phys..

[34] (2005). Amsterdam Density Functional (ADF) Program.

[35] Vosko S.H., Wilk L., Nusair M. (1980). Accurate spin-dependent electron liquid correlation energies for local spin density calculations: A critical analysis. Can. J. Phys..

[36] Versluis L., Ziegler T. (1988). The determination of molecular structures by density functional theory. The evaluation of analytical energy gradients by numerical integration. J. Chem. Phys..

